# Entropy Generation Analysis of Peristaltic Flow of Nanomaterial in a Rotating Medium through Generalized Complaint Walls of Micro-Channel with Radiation and Heat Flux Effects

**DOI:** 10.3390/mi13030375

**Published:** 2022-02-26

**Authors:** Aamir Ali, Mehak Sajid, Hafiz Junaid Anjum, Muhammad Awais, Kottakkaran Sooppy Nisar, C. Ahamed Saleel

**Affiliations:** 1 Department of Mathematics, Attock Campus, COMSATS University Islamabad, Kamra Road, Attock 43600, Pakistan; fa19-rmt-012@cuiatk.edu.pk (M.S.); awais@ciit-attock.edu.pk (M.A.); 2 Department of Mathematics, COMSATS University Islamabad, Park Road, Chak Shehzad, Islamabad 44000, Pakistan; mjunaidanjum@hotmail.com; 3 Department of Mathematics, College of Arts and Sciences, Prince Sattam bin Abdulaziz University, Wadi Aldawaser 11991, Saudi Arabia; 4 Department of Mechanical Engineering, College of Engineering, King Khalid University, Asir-Abha 61421, Saudi Arabia; ahamedsaleel@gmail.com

**Keywords:** entropy, peristaltic flow, heat flux, radiation, generalized complaint walls

## Abstract

This study discusses entropy generation analysis for a peristaltic flow in a rotating medium with generalized complaint walls. The goal of the current analysis is to understand the fluid flow phenomena particular to micro devices. Nano materials with a size less than 100 nm have applications in micro heat exchangers to cool electronic circuits, blood analyzers, biological cell separations, etc. For this study, we considered the effects of radiation, viscous dissipation and heat flux on the flow of nanomaterial inside a cylindrical micro-channel. To investigate the slip effects on the flow, the second order slip condition for axial velocity, the first order slip condition for secondary velocity and the thermal slip conditions were used. The flow was governed by partial differential equations (PDE’s), which were turned into a system of coupled ordinary differential equations (ODE’s) that were highly non-linear and numerically solved using the NDSolve command in Mathematica. The impacts of different involved parameters on the flow field were investigated with the aid of graphical illustrations. Entropy generation and the Bejan number were given special attention, and it was found that they decreased as the Hartman number, rotation, and radiation parameters increased.

## 1. Introduction

Peristalsis mainly refers to fluid flows driven by pressure gradients resulting from the movement of a wave along channel boundaries. The compression and expansion of the domain due to the propagation of the wave generates the flow. In the human body, urine flow, food transport through the digestive system, blood circulation (and others), all are due to peristalsis. Its application is also found in targeted drug delivery by the use of nano magnetic particles. The application of peristalsis can also be found in engineering, where it enables the construction of heart lung machines that operate on the principles of peristalsis. The study of thermal effects in peristaltic flows is motivated by its application to tumor growth, transportation of medical substances, such as a pill, or the transportation of nutrients to brain cells.

The research in this field can be traced back to pioneer works of Latham [1] and Shapiro et al. [2]. These studies provided a basic theoretical framework for investigating peristaltic flows with long wavelengths and low Reynolds numbers. Following their work, a great deal of research has been carried out by considering different flow configurations and a variety of Newtonian and non-Newtonian fluids. Yin et al. [3] studied the peristaltic flow of a viscous fluid in a circular tube. The mean flow and mean pressure gradients were reported to be proportionate with the square of the amplitude ratio in the absence of any wall motion. According to the numerical results, the mean axial velocity was dominated by the mean pressure gradient and the no-slip boundary condition. Asghar et al. [4] investigated the peristaltic flow of a reactive viscous fluid in a 2D setting, assuming viscosity to be temperature dependent. The existence of the Hartman layer, due to the presence of a magnetic field, and its consequent effects on the flow dynamics, were reported in [5]. Some other recent studies considering deforming walls and heat transfer effects in a peristaltic flow include [6,7,8,9] and references therein.

The issue of heat transfer arises in a wide range of engineering processes that use fluids such as refrigerants, water, engine oil, ethylene glycol, etc., as heat exchangers to maintain a desired working temperature. The poor thermal conductivity of the coolant fluids limits the effectiveness of these heat exchangers. It has been shown [10] that increasing the thermal conductivity of a fluid by introducing nanometer size particles into the base fluid used as a coolant can improve its thermal conductivity. The study of heat transfer in a peristaltic flow is motivated by its application in oxygenation and hemodialysis. Buongiorno [11] report their observations on convective transports in nanofluids, investigating various slip mechanisms attributed to the generation of relative velocity amongst the nanoparticles and the base fluid. Turkyilmazoglu [12] and Khan et al. [13] studied heat transfer effects in a nanofluid flow along stretching surfaces. Awais et al. [14] investigated the dynamical influence of nanoparticles on the flow of Oldroyd-B model polymeric liquids in the presence of nanoparticles. Other studies [15,16,17] consider flows in different geometrical configurations, investigating Brownian motions and thermophoresis effects on the flow dynamics and characteristics. In the existence of hydrodynamic slip and radiation impacts, Shashikumar et al. [18] conducted a thermodynamic study of Casson nanofluid in a porous microchannel. The Brownian motion and thermophoresis effects for Casson fluid on a stretched surface with non-Fourier heat flow were recently explored by Sreelakshmi et al. [19]. Fluid flow in small channels involving micro/nano materials holds importance in the study of micro ducts, micro pumps and valves, etc. Many scholars have studied these challenges in recent years as a result of these applications [20,21,22,23,24].

Complaint wall is defined as a wall that is deformable but is also stretchable, flexible and elastic in nature, with the ability to contain liquid in it. When a deforming force acts on this wall, it comes back to its original position due to its flexible and elastic nature. Elasticity is a characteristic of a body to restore itself once external deforming forces are removed, e.g., sponge, spring, rubber, etc. In contrast, plasticity is a material characteristic wherein material does not revert back to its original position when deforming force is removed, e.g., wood and glass. Deforming force is a force which brings change in shape, length or volume of the wall when applied. The amount of change is directly proportional to the force applied to the body. Every wall/medium has a limit to being elastic, which is termed as elastic limit; it means that a wall can bear a limited deformation force that is different for each medium to remain elastic, otherwise the deformation will be permanent. In complaint wall, all of these aspects are monitored. Movement of fluid is greatly dependent on complaint wall, as it manages the geometry of sinusoidal waves during peristalsis flow. The peristaltic flow of non-Newtonian fluid in a complaint walls channel was described by Ali et al. [25]. The analysis for MHD Jeffery fluid in a complaint walls channel was presented by Hayat et al. [26]. Heat transfer analysis of peristaltic flow in a complaint walls channel for viscous fluid was reported by Hayat et al. [27]. Nadeem et al. [28] discussed the trapping phenomenon in a rectangular channel with complaint walls for viscous fluid. The hall current investigations on peristaltic flow in a rotating medium were presented by Hayat et al. [29]. They considered the nanoparticles inside the channel of complaint walls. Recently, Awais et al. [30] presented the rheology of copper water nanomaterial in a channel by considering the generalized complaint walls and variable viscosity effects. In the branches of science, physiology and biology, magnetohydrodynamic (MHD) effect, which is critical for physiological liquids of peristaltic motion, has been discussed. Electric current causes a magnetic field due to the movement of conducting fluid particles changes in the fluid flow because of mechanical forces [31]. It has many applications in engineering, e.g., in aerodynamics, control of boundary layer, studies of plasma, energy removing from geothermal processes, MHD generator and inspection of oil; because of these applications, many inspectors focused on electrically conducting fluid passed through a porous medium with the magnetic field effects in peristalsis along transfer of mass and heat [32]. Kumar et al. [33] used the KKL model to investigate the effect of a magnetic dipole on the flow of a radiative nanofluid across a stretched surface. Alhumade et al. [34] examined the effects of nonlinear radiation on the flow over a stretched cylinder with the Cattaneo–Christove heat flux.

Heat transmission effects with the generation of entropy have been studied extensively since the pioneering work of Bejan [35], in which he presented a way to maximize the system’s destruction, heat-transmission effects with the formation of entropy. Entropy is a measure of arbitrariness inside a system or the degree of molecular disorder. In a thermodynamic system, entropy generation could result in a loss of energy attributed to various processes, e.g., friction, viscosity and chemical reactions. In diverse processes, counting chemical vapor testimony instruments, combustion, turbo machinery, electronic cooling devices, heat exchangers, and solar collectors, entropy plays a vital role. Entropy generation minimization is essential to increase the system performance in terms of heat conductivity. Hayat et al. [36] explored the entropy generation phenomenon for peristaltic flow in a rotating medium. The entropy generation investigation on magnetohydrodynamics peristaltic flow of copper–water nanofluid under slip effects was described by Ali et al. [37]. They claim that when slip parameters are used, entropy production diminishes. Shashikumar et al. [38] studied slip effects and performed an entropy generation study for the flow of a nanofluid in a microchannel employing aluminium and titanium alloy nanoparticles. Entropy-generating processes are investigated in a number of energy-related applications, including geothermal energy and modern refrigeration equipment [39,40,41,42,43,44].

As the entropy generation and compliant walls consideration can affect the flow dynamics and thermal characteristics for a peristaltic flow, we conducted this investigation to incorporate these effects, which were missing in the previously reported theoretical investigations. Motivated by this, the objective of this research was to analyze the entropy generation on the peristaltic flow in a rotating medium with generalized complaint walls. The entropy analysis was used to study the thermodynamic irreversibility, which destroys the available energy. Further, we also used nanoparticles in this analysis due to their vast engineering applications, as discussed above. The effects of radiation, viscous dissipation, and thermal flux on the flow of nanofluid inside a micro-channel are investigated. The second order slip condition for axial velocity, first order slip condition for secondary velocity, and thermal slip conditions are used to explore the effects of slip parameter on the flow. The flow was governed in non-dimensional form by highly non-linear PDE’s, which were then translated into a coupled system of ODE’s. Mathematica’s NDSolve tool was used to numerically solve the transformed system of coupled ODE’s. Graphs are used to explore the effects of several key parameters on the flow field.

## 2. Mathematical Modelling

Consider an unsteady peristaltic nanofluid flow in a porous, rotating medium with angular speed along the z−axis. 2d is the width of the channel with generalized complaint walls having temperatures $T_{1}\;\mathrm{and}\;T_{0}.$ The peristaltic flow arises due to the expansion of waves with wavelength λ, speed c, amplitude a, time t, and half channel width d aligned to the wall at z=±η expressed through the relation.

$$z=\pm \eta \left(x,t\right)=\pm \left[d+a\sin\left(\frac{2\pi }{\lambda }\right)\left(x-ct\right)\right].$$

**Figure 1:**
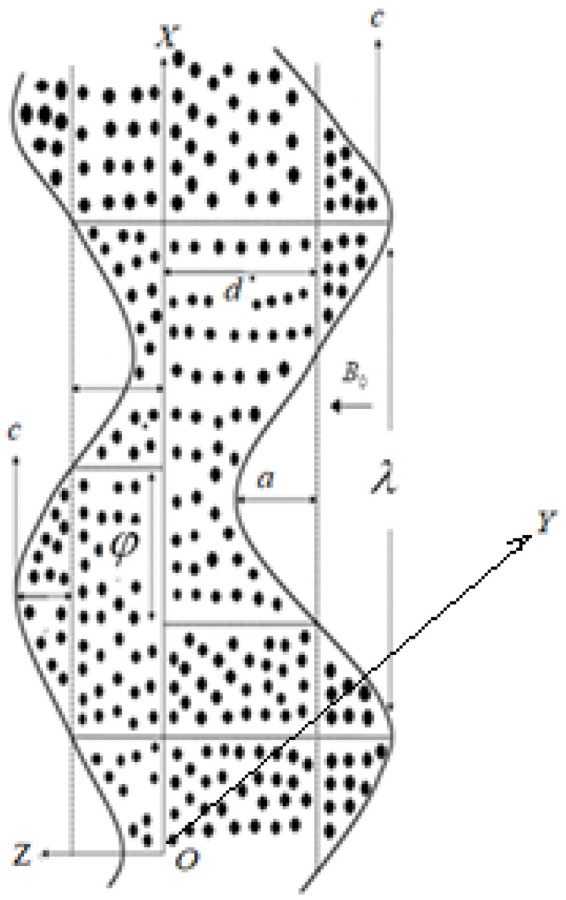
Schematic diagram of the problem.

Physically the problem is presented in Figure 1.

For incompressible flow, the mass, momentum and energy equations are [29,30]:

(1) ∇·V=0,

(2) $$\begin{array}{l} \rho_{nf}\left(\frac{dV}{dt}\right)+\rho_{nf}\left[\Omega \times \left(\Omega \times r\right)+2\left(\Omega \times V\right)\right]=\nabla \cdot \tau +J\times B-\frac{\mu_{nf}}{K}V\\ +g{\left(\rho \beta \right)}_{nf}\left(T-T_{m}\right), \end{array}$$

(3) $${\left(\rho c_{p}\right)}_{nf}\left(\frac{dT}{dt}\right)=\kappa_{nf}\nabla^{2}T+\tau \cdot L-\nabla q_{r}-\frac{1}{\sigma_{nf}}J\cdot J+Q_{0},$$

where, $\frac{d}{dt}$ is material time derivative, V=V[u(x,z,t),v(x,z,t),w(x,z,t)] is velocity field, $\rho_{nf}$ is nanofluid density, $\Omega =\Omega \hat{k}$ is angular velocity, τ is Cauchy stress tensor, B is magnetic field, J is current density, J×B is Lorentz force, $\mu_{nf}$ is nanofluid viscosity, ${\left(\rho \beta \right)}_{nf}$ is coefficient of thermal expansion, ${\left(\rho c_{p}\right)}_{nf}$ is heat capacity of nanofluid, $\kappa_{nf}$ is thermal conductivity of nanofluid, T is temperature of fluid, τ·L is viscous dissipation term, $q_{r}$ is thermal radiation term, ${\left(\sigma \right)}_{nf}$ is electric conductivity of nanofluid, J·J is joule heating term, and $Q_{0}$ is heat generation/absorption parameter. The Cauchy stress tensor is expressed as:

(4) $$\tau =-PI+\mu_{nf}A,$$

where P is pressure. A is Rivilin Ericksen tensor and is defined by the relation:

$$A=\left(\nabla \cdot V\right)+{\left(\nabla \cdot V\right)}^{t}.$$

The modified Ohm’s law is given as:

(5) $$J=\sigma_{nf}\left[E+\left(V\times B\right)-\frac{1}{en_{e}}\left(J\times B\right)\right],$$

In above equations, $\frac{1}{en_{e}}$ is the hall factor where e the electron charge and $n_{e}$ is the density of free electron. When there is electric field E, the Lorentz force becomes:

(6) $$J\times B=[\frac{\sigma_{nf}B_{0}^{2}}{1+{\left(\frac{\sigma_{nf}B_{0}}{en_{e}}\right)}^{2}}\left(-u+\left(\frac{\sigma_{nf}B_{0}}{en_{e}}\right)v\right),-\frac{\sigma_{nf}B_{0}^{2}}{1+{\left(\frac{\sigma_{nf}B_{0}}{en_{e}}\right)}^{2}}\left(v+\left(\frac{\sigma_{nf}B_{0}}{en_{e}}\right)u\right),0].$$

The expression for rotational force is:

(7) $$\Omega \times \left(\Omega \times r\right)+2\left(\Omega \times V\right)=-\Omega \left(\Omega x+2v\right)\hat{i}-\Omega \left(\Omega y-2u\right)\hat{j}+0\hat{k}.$$

The radiative diffusion in the Rosselend approximation is defined as:

(8) $$q_{r}=-\frac{4\sigma^{*}}{3k^{*}}\frac{\partial T^{4}}{\partial x},$$

where $\sigma^{*}$ is the Stefan–Boltzmann constant, whereas $k^{*}$ is the mean absorption coefficient. When the Taylor series expansion is applied to the $T^{4}$ about mean temperature of nano-material $T_{w}$, we get:

(9) $$\nabla q_{r}=\frac{\partial q_{r}}{\partial x}+\frac{\partial q_{r}}{\partial z}=-\frac{16\sigma^{*}T_{m}^{3}}{3k^{*}}\left[\frac{\partial^{2}T}{\partial x^{2}}+\frac{\partial^{2}T}{\partial z^{2}}\right].$$

The relation of viscous dissipation is:

(10) $$\tau \cdot L=\mu_{nf}[2\{{\left(\frac{\partial u}{\partial x}\right)}^{2}+{\left(\frac{\partial w}{\partial z}\right)}^{2}\}+({\frac{\partial u}{\partial z}+\frac{\partial w}{\partial x})}^{2}].$$

We obtain the following system of equations in components form by substituting Equations (4), (6), (7), (9) and (10) into fundamental Equations (1)–(3):

(11) $$\frac{\partial u}{\partial x}+\frac{\partial w}{\partial z}=0,$$

(12) $$\begin{array}{l} \rho_{nf}\left(\frac{du}{dt}-2\Omega v\right)=-\frac{\partial \hat{P}}{\partial x}+\mu_{nf}\left(\frac{\partial^{2}u}{\partial x^{2}}+\frac{\partial^{2}u}{\partial z^{2}}\right)+\frac{\sigma_{nf}B_{0}^{2}}{1+{\left(\frac{\sigma_{nf}B_{0}}{en_{e}}\right)}^{2}}\left(-u+\left(\frac{\sigma_{nf}B_{0}}{en_{e}}\right)v\right)\\ -\frac{\mu_{nf}}{K}u+g{\left(\rho \beta \right)}_{nf}\left(T-T_{m}\right), \end{array}$$

(13) $$\begin{array}{l} \rho_{nf}\left(\frac{dv}{dt}+2\Omega u\right)=-\frac{\partial \hat{P}}{\partial y}+\mu_{nf}\left(\frac{\partial^{2}v}{\partial x^{2}}+\frac{\partial^{2}v}{\partial z^{2}}\right)-\frac{\sigma_{nf}B_{0}^{2}}{1+{\left(\frac{\sigma_{nf}B_{0}}{en_{e}}\right)}^{2}}\left(v+\left(\frac{\sigma_{nf}B_{0}}{en_{e}}\right)u\right)\\ -\frac{\mu_{nf}}{K}v, \end{array}$$

(14) $$\rho_{nf}\frac{dw}{dt}=-\frac{\partial \hat{P}}{\partial z}+\mu_{nf}\left(\frac{\partial^{2}w}{\partial x^{2}}+\frac{\partial^{2}w}{\partial z^{2}}\right),$$

(15) $$\begin{array}{l} {\left(\rho c_{p}\right)}_{nf}\frac{dT}{dt}=\kappa_{nf}\left(\frac{\partial^{2}T}{\partial x^{2}}+\frac{\partial^{2}T}{\partial z^{2}}\right)+\mu_{nf}\left[2\left\{{\left(\frac{\partial u}{\partial x}\right)}^{2}+{\left(\frac{\partial w}{\partial z}\right)}^{2}\right\}+{\left(\frac{\partial u}{\partial z}+\frac{\partial w}{\partial x}\right)}^{2}\right]\\ +\frac{16\sigma^{*}T_{m}^{3}}{3k^{*}}\left[\frac{\partial^{2}T}{\partial x^{2}}+\frac{\partial^{2}T}{\partial z^{2}}\right]+\frac{\mu_{nf}}{K}u^{2}+\Phi . \end{array}$$

The centrifugal effect with modified pressure $\hat{P}$ can be expressed as:

(16) $$\hat{P}=p-\frac{1}{2}\rho \Omega^{2}\left(x^{2}+y^{2}\right).$$

The values for $\rho_{nf},\mu_{nf},\;{\left(\rho c_{p}\right)}_{nf},\kappa_{nf},\;{\left(\rho \beta \right)}_{nf}\mathrm{and}\;\sigma_{nf}$ are presented in Table 1 where subscript p denotes particle of copper and f for base fluid. The numerical values of these quantities for nanoparticles has been provided in Table 2.

### 2.1. Thermo-Physical Properties

The important relations for nano-material fluid are given in Table 1.

The expression for generalized complaint wall is:

(17) $$L\left(\eta \right)=P-P_{0}$$

and

(18) $$L\left(\eta \right)=\left[-\tau \frac{\partial^{2}}{\partial x^{2}}+m^{\prime }\frac{\partial^{2}}{\partial t^{2}}+d^{\prime }\frac{\partial }{\partial t}+\beta^{\prime }\frac{\partial^{4}}{\partial x^{4}}+k\right]\eta ,$$

where, τ is the wall velocity, $m^{\prime }$ is the plate mass, $d^{\prime }$ is the wall-damping coefficient, $\beta^{\prime }$ is the stiffness in flexure, k is the stiffness effects.

$$\frac{\partial L}{\partial x}=\frac{\partial p}{\partial x}$$

(19) $$\begin{array}{l} \frac{\partial L}{\partial x}=\mu_{nf}\left(\frac{\partial^{2}u}{\partial x^{2}}+\frac{\partial^{2}u}{\partial z^{2}}\right)+\frac{\sigma_{nf}B_{0}^{2}}{1+{\left(\frac{\sigma_{nf}B_{0}}{en_{e}}\right)}^{2}}\left(-u+\left(\frac{\sigma_{nf}B_{0}}{en_{e}}\right)v\right)\\ -\frac{\mu_{nf}}{K}u+g{\left(\rho \beta \right)}_{nf}\left(T-T_{m}\right)-\rho_{nf}\left(\frac{du}{dt}-2\Omega v\right). \end{array}$$

To convert Equations (11)–(15) into non-dimensional form, we utilize the following set of non-dimensional variables:

(20) x*=xλ,  y*=yλ,  z*=zd,  p*=d2P^cμλ,  t*=ctλ,  u*=uc,  v*=vc,w*=wc,  η*=ηd,  δ=dλ,  Tm=T1−T02,  θ=T−TmT1−T0,  K1=Kd2,T*=ReΩdc,   u=∂ψ∂z,  w=−δ∂ψ∂x.

where ψ is the stream function, $T_{m}$ is the mean temperature, $T_{1}$ is the upper wall temperature, $T_{0}$ is the lower wall temperature. Utilizing these non-dimensional variables and stream function defined in (20) into Equations (11)–(15), the continuity Equation (11) is satisfied identically, while momentum and heat equations becomes:

(21) $$\begin{array}{l} Re\delta \left(\frac{\rho_{nf}}{\rho_{f}}\right)\frac{d}{dt}\left(\frac{\partial \psi }{\partial z}\right)-2T^{\prime }\left(\frac{\rho_{nf}}{\rho_{f}}\right)v=-\frac{\partial p}{\partial x}+Gr\left(\frac{{\left(\rho c_{p}\right)}_{nf}}{{\left(\rho c_{p}\right)}_{f}}\right)\theta \\ +\left(\frac{\mu_{nf}}{\mu_{f}}\right)\left[\delta^{2}\frac{\partial^{3}\psi }{\partial x^{2}\partial z}+\frac{\partial^{3}\psi }{\partial z^{3}}-\frac{1}{K_{1}}\frac{\partial \psi }{\partial z}\right]-\frac{\left(\frac{\sigma_{nf}}{\sigma_{f}}\right)M^{2}}{1+{\left(\left(\frac{\sigma_{nf}}{\sigma_{f}}\right)m\right)}^{2}}\left(\frac{\partial \psi }{\partial z}-\left(\frac{\sigma_{nf}}{\sigma_{f}}\right)mv\right),\; \end{array}$$

(22) $$\begin{array}{l} Re\delta \left(\frac{\rho_{nf}}{\rho_{f}}\right)\frac{dv}{dt}+2T^{\prime }\left(\frac{\rho_{nf}}{\rho_{f}}\right)\frac{\partial \psi }{\partial z}=-\frac{\partial p}{\partial y}\\ +\left(\frac{\mu_{nf}}{\mu_{f}}\right)\left[\delta^{2}\frac{\partial^{2}\psi }{\partial x^{2}}+\frac{\partial^{2}v}{\partial z^{2}}-\frac{v}{K_{1}}\right]-\frac{\left(\frac{\sigma_{nf}}{\sigma_{f}}\right)M^{2}}{1+{\left(\left(\frac{\sigma_{nf}}{\sigma_{f}}\right)m\right)}^{2}}\left(v+A_{1}m\frac{\partial \psi }{\partial z}\right), \end{array}$$

(23) $$-Re\delta^{2}\left(\frac{\rho_{nf}}{\rho_{f}}\right)\frac{d}{dt}\left(\frac{\partial \psi }{\partial x}\right)=-\frac{\partial p}{\partial z}+\left(\frac{\mu_{nf}}{\mu_{f}}\right)\left[\delta^{3}\frac{\partial^{3}\psi }{\partial x^{3}}-\delta \frac{\partial^{3}\psi }{\partial z^{2}\partial x}\right],\;$$

(24) δRePr(ρnfρf)[∂θ∂t+∂ψ∂z∂θ∂x+v∂θ∂y−∂ψ∂x∂θ∂z]=Gr(ρβ)nf(ρβ)f[δ2∂2θ∂x2+∂2θ∂z2]+(μnfμf)BrK1(∂ψ∂z)2+4Rd3[δ2∂2θ∂x2+∂2θ∂z2]+ε1+(μnfμf)Br[2δ2{(∂2ψ∂x∂z)2+(∂2ψ∂z2)2}+(∂2ψ∂z2−δ2∂2ψ∂x2)2].

In above non-dimensional Equations (21)–(24), M, Re, Ec, Pr, Rd, $\varepsilon_{1},$ m, Gr, and Br are the Hartman number, the Reynolds number, the Eckert number, the Prandtl number, the radiation parameter, the heat generation/absorption parameter, the Hall parameter, the Grashof number, and the Brinkman number, respectively, which are non-dimensional parameters and are mathematically defined as:

(25) M=B0dσfμf,Re=ρfcdμf,Ec=d2μf(cp)f(T1−T0),Pr=μf(cp)fκf,Rd=4σ*Tm3k*κf,ε1=d2Φκf(T1−T0), m=σfB0ene,Gr=g(ρβ)fd2cμf(T1−T0),Br=EcPr.

We get the following simplified form of Equations (21)–(24), when we apply the assumption of long wavelength and modest inertial forces to momentum and energy equations:

(26) $$\begin{array}{l} -2T^{\prime }\left(\frac{\rho_{nf}}{\rho_{f}}\right)v=-\frac{\partial p}{\partial x}+\left(\frac{\mu_{nf}}{\mu_{f}}\right)\left[\frac{\partial^{3}\psi }{\partial z^{3}}-\frac{1}{K_{1}}\frac{\partial \psi }{\partial z}\right]\\ -\frac{\left(\frac{\sigma_{nf}}{\sigma_{f}}\right)M^{2}}{1+{\left(\left(\frac{\sigma_{nf}}{\sigma_{f}}\right)m\right)}^{2}}\left(\frac{\partial \psi }{\partial z}-\left(\frac{\sigma_{nf}}{\sigma_{f}}\right)mv\right)+Gr\left(\frac{{\left(\rho c_{p}\right)}_{nf}}{{\left(\rho c_{p}\right)}_{f}}\right)\theta ,\; \end{array}$$

(27) $$2T^{\prime }\left(\frac{\rho_{nf}}{\rho_{f}}\right)\frac{\partial \psi }{\partial z}=-\frac{\partial p}{\partial y}+\left(\frac{\mu_{nf}}{\mu_{f}}\right)\left[\frac{\partial^{2}v}{\partial z^{2}}-\frac{v}{K_{1}}\right]-\frac{\left(\frac{\sigma_{nf}}{\sigma_{f}}\right)M^{2}}{1+{\left(\left(\frac{\sigma_{nf}}{\sigma_{f}}\right)m\right)}^{2}}\left(v+A_{1}m\frac{\partial \psi }{\partial z}\right),$$

(28) $$\frac{\partial p}{\partial z}=0,\;$$

(29) $$\begin{array}{l} Gr\frac{{\left(\rho \beta \right)}_{nf}}{\left(\rho \beta \right)f}\left[\frac{\partial^{2}\theta }{\partial z^{2}}\right]+\frac{4Rd}{3}\left[\frac{\partial^{2}\theta }{\partial z^{2}}\right]+\left(\frac{\mu_{nf}}{\mu_{f}}\right)\frac{Br}{K_{1}}{\left(\frac{\partial \psi }{\partial z}\right)}^{2}\\ +\varepsilon_{1}+\left(\frac{\mu_{nf}}{\mu_{f}}\right)Br{\left(\frac{\partial^{2}\psi }{\partial z^{2}}\right)}^{2}=0. \end{array}$$

Now, η amplitude ratio parameter with wall properties gives non-dimensional boundary conditions:

(30) $$\begin{array}{ll} \frac{\partial \psi }{\partial z}\pm \alpha_{1}\left(\frac{\mu_{nf}}{\mu_{f}}\right)\frac{\partial^{2}\psi }{\partial z^{2}}\pm \alpha_{2}\left(\frac{\mu_{nf}}{\mu_{f}}\right)\frac{\partial^{3}\psi }{\partial z^{3}}=0 & \mathrm{at}\;z=\pm \eta \\ \left[E_{1}\frac{\partial^{3}}{\partial x^{3}}+E_{2}\frac{\partial^{3}}{\partial x\partial t^{2}}+E_{3}\frac{\partial^{2}}{\partial x\partial t}+E_{4}\frac{\partial^{5}}{\partial x^{5}}+E_{5}\frac{\partial }{\partial x}\right]\eta =\frac{\partial p}{\partial x} & \mathrm{at}\;z=\pm \eta \\ v\pm \beta_{1}\left(\frac{\mu_{nf}}{\mu_{f}}\right)\left[\frac{\partial v}{\partial z}\right]=0 & \mathrm{at}\;z=\pm \eta \\ \theta \pm \beta_{2}\left(\frac{\mu_{nf}}{\mu_{f}}\right)\left[\frac{\partial \theta }{\partial z}\right]=\pm \frac{1}{2} & \mathrm{at}\;z=\pm \eta \end{array}$$

Here, $\alpha_{1},\alpha_{2},\beta_{1},\;\mathrm{and}\;\beta_{2}$ are first order, second order, secondary velocity slip, and thermal slip parameters. The main aim was to eliminate the pressure from x and y components of the momentum equation; thus, the author used Equation (28), which already represented that pressure does not depends on z. Secondary flow is due to rotation, so pressure can be ignored from Equation (27). Thus, we have

(31) $$\begin{array}{l} \left(\frac{\mu_{nf}}{\mu_{f}}\right)\left[\frac{\partial^{4}\psi }{\partial z^{4}}-\frac{1}{K_{1}}\frac{\partial^{2}\psi }{\partial z^{2}}\right]-\frac{\left(\frac{\sigma_{nf}}{\sigma_{f}}\right)M^{2}}{1+{\left(\left(\frac{\sigma_{nf}}{\sigma_{f}}\right)m\right)}^{2}}\left(\frac{\partial^{2}\psi }{\partial z^{2}}-\left(\frac{\sigma_{nf}}{\sigma_{f}}\right)m\frac{\partial v}{\partial z}\right)\\ +2T^{\prime }\left(\frac{\rho_{nf}}{\rho_{f}}\right)\frac{\partial v}{\partial z}+Gr\left(\frac{{\left(\rho c_{p}\right)}_{nf}}{{\left(\rho c_{p}\right)}_{f}}\right)\frac{\partial \theta }{\partial z}=0,\; \end{array}$$

(32) $$\left(\frac{\mu_{nf}}{\mu_{f}}\right)\left[\frac{\partial^{2}v}{\partial z^{2}}-\frac{v}{K_{1}}\right]-2T^{\prime }\left(\frac{\rho_{nf}}{\rho_{f}}\right)\frac{\partial \psi }{\partial z}-\frac{\left(\frac{\sigma_{nf}}{\sigma_{f}}\right)M^{2}}{1+{\left(\left(\frac{\sigma_{nf}}{\sigma_{f}}\right)m\right)}^{2}}\left(v+\left(\frac{\sigma_{nf}}{\sigma_{f}}\right)m\frac{\partial \psi }{\partial z}\right)=0,$$

At the end the simplified form of non-dimensional equations with boundary conditions is:

(33) $$A_{0}\left(\frac{\partial^{4}\psi }{\partial z^{4}}-\frac{1}{K_{1}}\frac{\partial^{2}\psi }{\partial z^{2}}\right)-\frac{A_{1}M^{2}}{1+{\left(A_{1}m\right)}^{2}}\left(\frac{\partial^{2}\psi }{\partial z^{2}}-A_{1}m\frac{\partial v}{\partial z}\right)+2T^{\prime }A_{2}\frac{\partial v}{\partial z}+A_{3}\frac{\partial \theta }{\partial z}=0,$$

(34) $$A_{0}\left(\frac{\partial^{2}v}{\partial z^{2}}-\frac{v}{K_{1}}\right)-\frac{A_{1}M^{2}}{1+{\left(A_{1}m\right)}^{2}}\left(v+A_{1}m\frac{\partial \psi }{\partial z}\right)-2T^{\prime }A_{2}\frac{\partial \psi }{\partial z}=0,$$

(35) $$\left(A_{4}+\frac{4}{3}Rd\right)\frac{\partial^{2}\theta }{\partial z^{2}}+A_{0}\frac{Br}{K_{1}}{\left(\frac{\partial \psi }{\partial z}\right)}^{2}+A_{0}Br{\left(\frac{\partial^{2}\psi }{\partial z^{2}}\right)}^{2}+\varepsilon_{1}=0.$$

With boundary conditions:

(36) $$\begin{array}{ll} \frac{\partial \psi }{\partial z}\pm \alpha_{1}A_{0}\frac{\partial^{2}\psi }{\partial z^{2}}\pm \alpha_{2}A_{0}\frac{\partial^{3}\psi }{\partial z^{3}}=0 & \mathrm{at}\;z=\pm \eta \\ \left[E_{1}\frac{\partial^{3}}{\partial x^{3}}+E_{2}\frac{\partial^{3}}{\partial x\partial t^{2}}+E_{3}\frac{\partial^{2}}{\partial x\partial t}+E_{4}\frac{\partial^{5}}{\partial x^{5}}+E_{5}\frac{\partial }{\partial x}\right]\eta =\frac{\partial p}{\partial x} & \mathrm{at}\;z=\pm \eta \\ v\pm \beta_{1}A_{0}\frac{\partial v}{\partial z}=0 & \mathrm{at}\;z=\pm \eta \\ \theta \pm \beta_{2}A_{0}\frac{\partial \theta }{\partial z}=\pm \frac{1}{2} & \mathrm{at}\;z=\pm \eta \end{array}$$

In the above equations, the constants that are used are given below:

(37) $$\begin{array}{l} A_{0}=\frac{\mu_{nf}}{\mu_{f}},\;A_{1}=\frac{\sigma_{nf}}{\sigma_{f}},\;A_{2}=\frac{\rho_{nf}}{\rho_{f}},\;A_{3}=Gr\cdot \frac{{\left(\rho c_{p}\right)}_{nf}}{{\left(\rho c_{p}\right)}_{f}},\;A_{4}=\frac{{\left(\rho \beta \right)}_{nf}}{{\left(\rho \beta \right)}_{f}},\\ E_{1}=-\frac{{}_{\tau d^{3}}}{\lambda^{3}\mu_{f}c},E_{2}=\frac{{}_{m_{1}cd^{3}}}{\lambda^{3}\mu_{f}},E3=\frac{{}_{d^{\prime }d^{3}}}{\lambda^{2}\mu_{f}},E_{4}=\frac{{}_{\beta^{\prime }d^{3}}}{\lambda^{3}\mu_{f}c},E_{5}=\frac{{}_{kd^{3}}}{\lambda \mu_{f}c}. \end{array}$$

### 2.2. Entropy Generation Analysis

An irreversible process in which two phenomena occur, i.e., thermal diffusion and fluid friction, results in entropy generation, which is basically loss of ability to do work. The volume fraction entropy generation for two-phase nanomaterial is:

(38) $$\begin{array}{l} Ngen=\frac{\kappa_{nf}}{\theta_{0}^{2}}\left[{\left(\frac{\partial T}{\partial x}\right)}^{2}+{\left(\frac{\partial T}{\partial z}\right)}^{2}\right]+\frac{\mu_{nf}}{\theta_{0}}\left[2{\left(\frac{\partial u}{\partial x}\right)}^{2}+{\left(\frac{\partial w}{\partial z}\right)}^{2}+{\left(\frac{\partial u}{\partial z}+\frac{\partial w}{\partial x}\right)}^{2}\right]\\ +\frac{16\sigma^{*}T_{m}^{3}}{3k^{*}\theta_{0}}\left[\frac{\partial^{2}T}{\partial x^{2}}+\frac{\partial^{2}T}{\partial z^{2}}\right]+\frac{\mu_{nf}u^{2}}{K\theta_{0}}+\frac{\Phi }{\theta_{0}}. \end{array}$$

The first term on the right side is irreversible heat transfer, the second term is irreversible viscous dissipation, the third term is radiation effects, the fourth term is heat transfer analysis for convection, and the fifth term is heat generation/absorption effects. The entropy generation number in non-dimensional form is:

(39) $$N_{s}=\frac{N_{gen}}{N_{g}}=\left(A_{4}+\frac{4}{3}Rd\right){\left(\frac{\partial \theta }{\partial z}\right)}^{2}+A_{0}Br\Lambda \left[\frac{1}{K_{1}}{\left(\frac{\partial \psi }{\partial z}\right)}^{2}+{\left(\frac{\partial^{2}\psi }{\partial z^{2}}\right)}^{2}\right]+\varepsilon_{1}$$

where Ng is the rate of entropy generation and Λ is the temperature ratio, defined as:

(40) $$N_{g}=\frac{\kappa_{f}{\left(T_{1}-T_{0}\right)}^{2}}{\theta_{0}^{2}d^{2}},\Lambda =\frac{\theta_{0}}{\left(T_{1}-T_{0}\right)}$$

### 2.3. Bejan Number Analysis

The Bejan number was introduced by professor Adrian Bejan from Duke University. The Bejan number is the proportion of heat transfer irreversibility to total entropy generation. Mathematically,

(41) $$Be=\frac{\left(A4+\frac{4}{3}Rd\right){\left(\frac{\partial \theta }{\partial z}\right)}^{2}}{\left(A_{4}+\frac{4}{3}Rd\right){\left(\frac{\partial \theta }{\partial z}\right)}^{2}+A_{0}Br\Lambda \left[\frac{1}{K_{1}}{\left(\frac{\partial \psi }{\partial z}\right)}^{2}+{\left(\frac{\partial^{2}\psi }{\partial z^{2}}\right)}^{2}\right]+\varepsilon_{1}}$$

The variation of the Bejan number is 0<Be<1, which implies that total entropy generation dominates heat transfer irreversibility in one case and that total entropy generation equals heat transfer irreversibility in the other.

## 3. Graphical Discussion

The focus of this work is on the analysis of entropy generation of dual-stage nanomaterial in a peristaltic motion considering thermal fluxes and radiation, along with the boundary condition of the generalized complaint wall in a rotating channel. The governing equations, derived in the preceding section, were solved in Mathematica using built-in solver NDSolve. In this section, we discuss the physical impacts of various parameters (Hall parameter (m), radiation parameter (Rd), permeability parameter ($K_{1}$), heat generation/absorption parameter ($\varepsilon_{1}$), Hartman number (M), rotation parameter ($T^{\prime }$), first and second order velocity slip parameters ($\alpha_{1},\alpha_{2}$), secondary velocity slip ($\beta_{1}$), and thermal slip parameters ($\beta_{2}$) on axial and secondary velocities (u and v), temperature distribution (θ), entropy production (Ns), and Bejan number (Be) for the given values of t=0.1, x=0.2, ϕ=0.01, ε=0.3, $E_{1}=0.03,$ $E_{2}=0.02,$ $E_{3}=0.01,$ $E_{4}=0.03,$ $E_{5}=0.02$).

### 3.1. Axial Velocity Analysis

**Figure 2:**
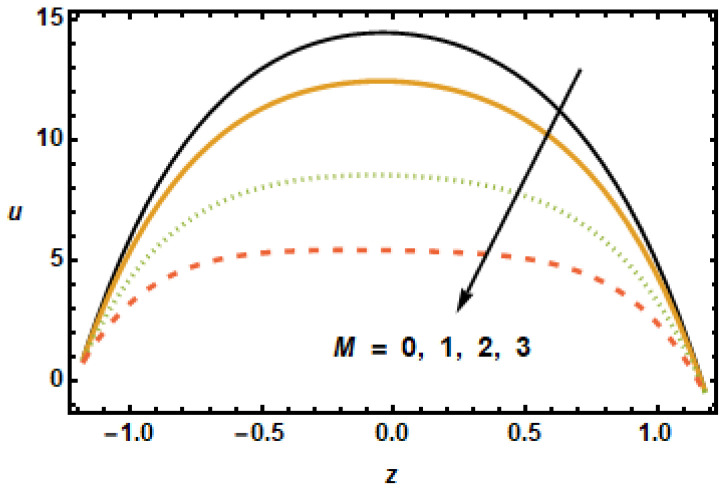
Effects of Hartmann number M on u.

**Figure 3:**
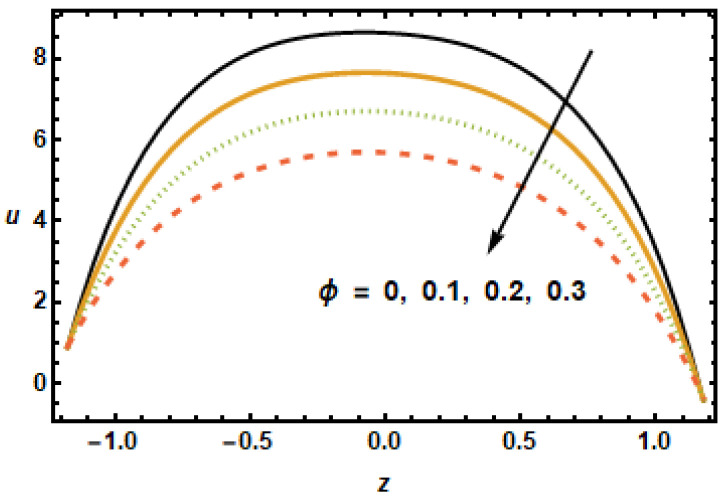
Effects of Volume fraction ϕ on u.

**Figure 4:**
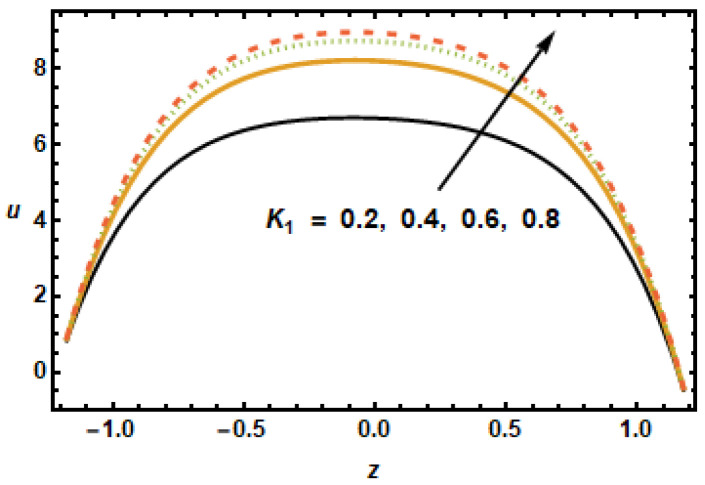
Effects of permeability $K_{1}$ on u.

**Figure 5:**
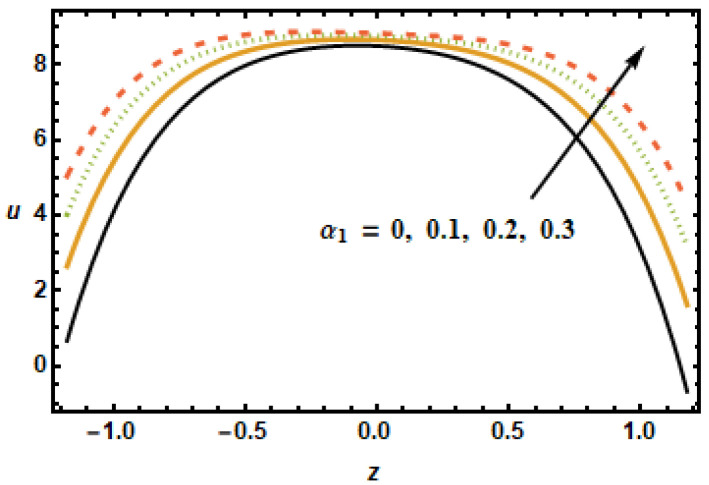
Effects of first order slip $\alpha_{1}$ on u.

**Figure 6:**
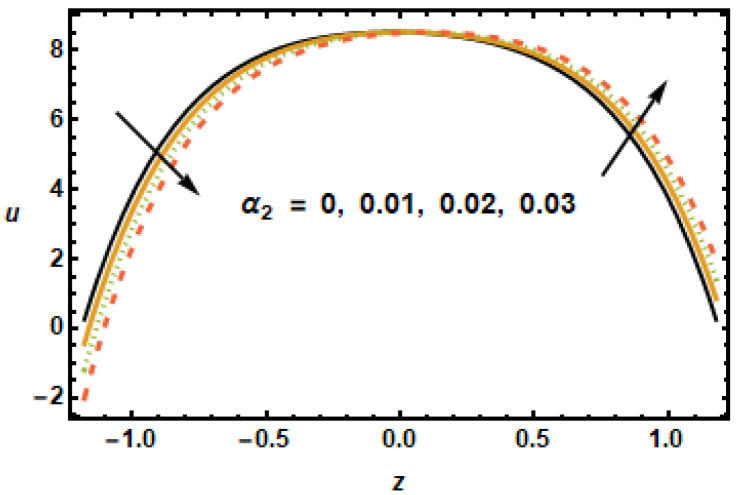
Effects of second order slip $\alpha_{2}$ on u.

**Figure 7:**
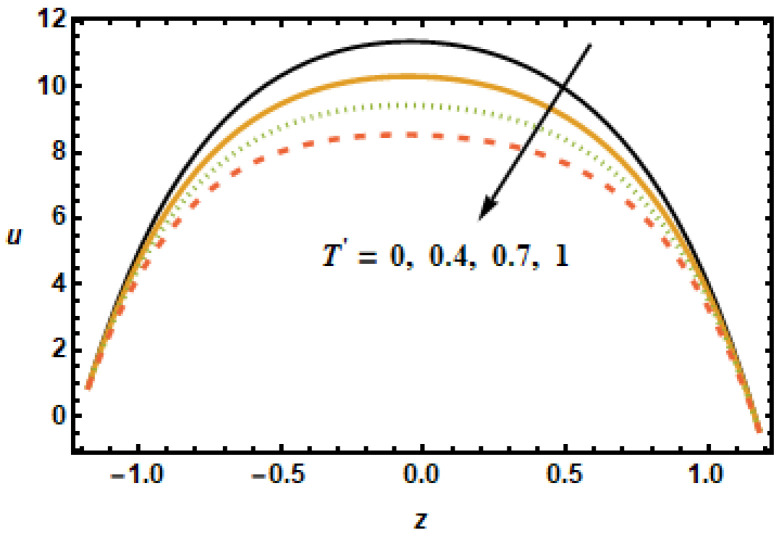
Effects of rotation parameter $T^{\prime }$ on u.

The effects of various flow parameters, such as Hartman number (M), nanoparticles volume fraction (ϕ), permeability parameter ($K_{1}$), first order velocity slip ($\alpha_{1}$), second order velocity slip ($\alpha_{2}$) and rotation parameter ($T^{\prime }$) on the axial velocity *u* are presented in Figure 2, Figure 3, Figure 4, Figure 5, Figure 6 and Figure 7. The results are calculated for ε=0.3, m=1, Gr=3, Rd=0.1, Br=0.01, $K_{1}=0.5,$ M=2, $\beta_{1}=0.02,$ $\beta_{2}=0.02,$ $\alpha_{1}=0.01,$ and $\beta_{1}=-0.01.$ Figure 2 shows curves representing spatial distribution of axial velocities *u* computed for several Hartman numbers *M*, ranging from 0 to 3. It is noted that, as the Hartmann number increased, the axial velocity decreased. Furthermore, the velocity gradients in the interior domain also decreased as M increased. These observations are consistent with the physical characteristics of magnetic force, i.e., Lorentz force is a decelerating force, and also with the previously reported experimental and theoretical observations. Figure 3 depicts that the axial velocity decreased with the increase in the values of volume fraction ϕ, due to strong resistive forces. The small change was observed at the starting and end points; however, at the middle of the graph, axial velocity distribution had great loss.

Figure 4 shows axial velocity plots for numerous values of permeability parameter $K_{1}$, representing the permeability of the porous medium. The plotted results show that the axial velocity rose with increasing values of the permeability parameter since, with increased permeability of porous medium $K_{1}$, frictional forces decreased, hence, the fluid accelerated because of higher energy budgets. The effects of slip parameter $\alpha_{1}$ on the axial velocity distributions are shown in Figure 5. The results plotted in the figure, for different values of $\alpha_{1}$, shows that the slip parameter affects flow dynamics in the locality of the boundary as expected. It is noted that increase in slip parameter $\alpha_{1}$ had accelerating effects on the axial velocity; therefore, axial velocity increased with increasing $\alpha_{1}$. When the slip effects were incorporated in the applied boundary condition, the energy losses at the boundaries decreased, which is why the slip parameter $\alpha_{1}$ exhibited accelerating effects on the flow dynamics.

Similar observations are made in Figure 6, showing axial velocity for different values of second slip parameter $\alpha_{2}$. In contrast to $\alpha_{1}$, the second slip parameter $\alpha_{2}$ is linked with the spatial change in the velocity gradients. It is, therefore, seen in plotted results that the axial velocities decreased in the lower half, since velocity gradients were decreasing in *z*, and increased in the upper half due to increasing velocity gradients. In Figure 7, we show axial velocities for different values of the rotation parameter $T^{\prime }$. The results show that the increase in the values of rotational parameter $T^{\prime }$ resulted in a decrease in axial velocity, exhibiting an inverse relation between the velocity and rotation. Notice that the maximum velocity in the axial direction was achieved when rotation was zero, as, in that case, the retarding effects of the magnetic field were minimal.

### 3.2. Secondary Velocity Analysis

**Figure 8:**
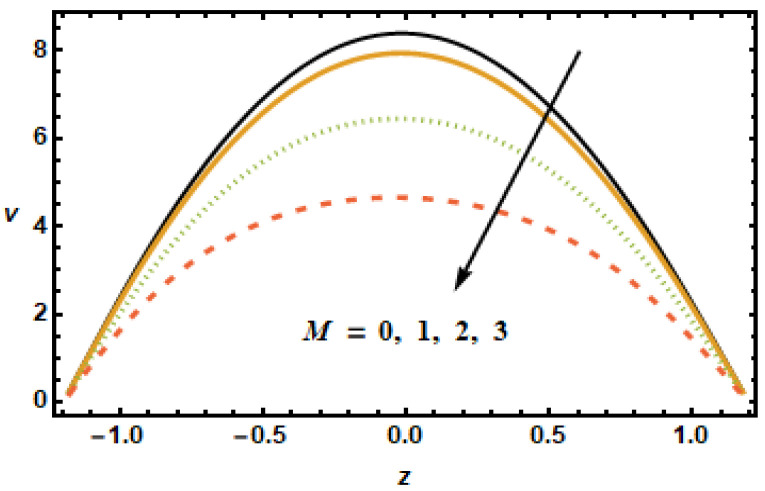
Dependence of v on Hartman number M.

**Figure 9:**
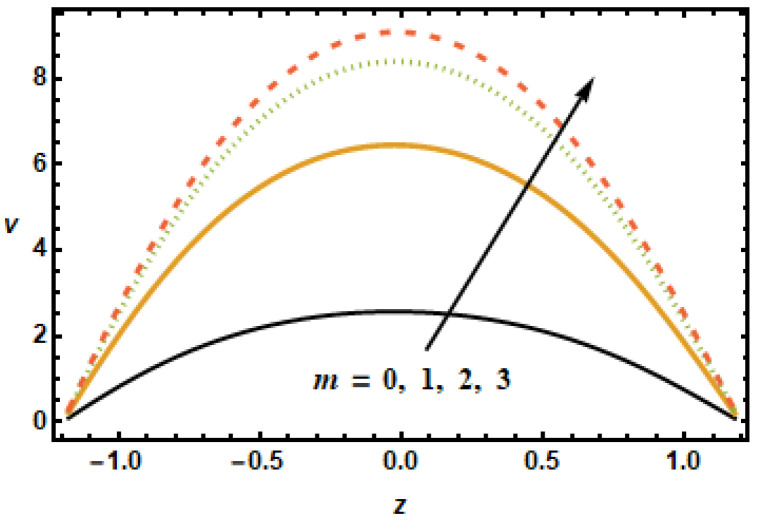
Dependence of v on Hartman number m.

**Figure 10:**
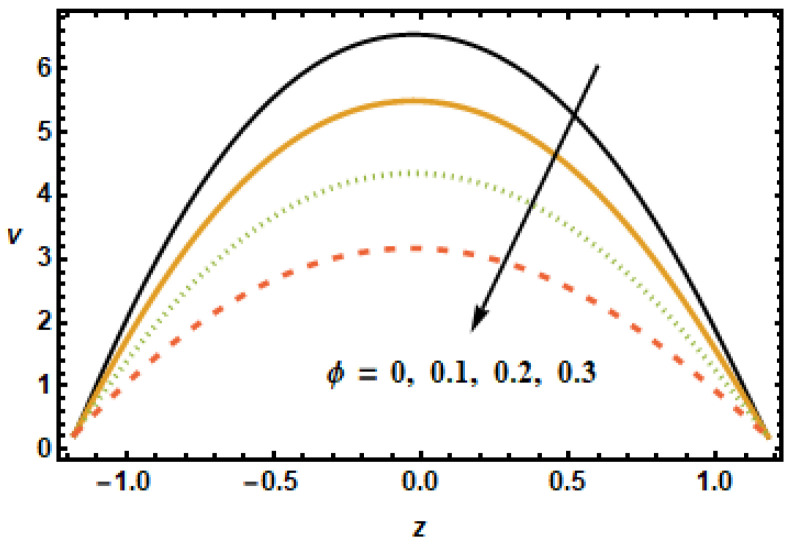
Dependence of v on volume fraction ϕ.

**Figure 11:**
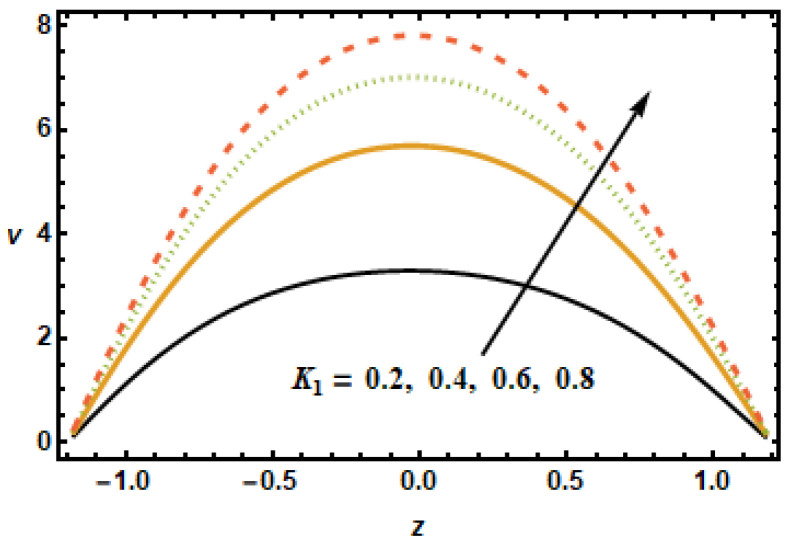
Dependence of v on permeability $K_{1}$.

**Figure 12:**
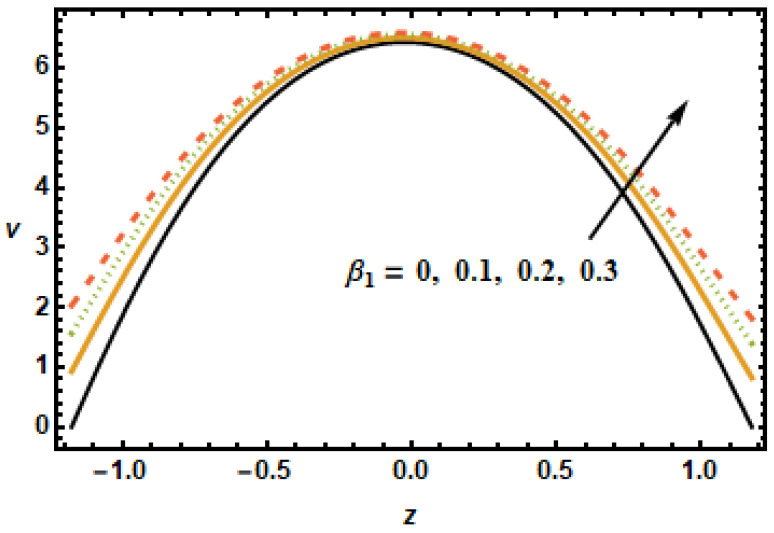
Dependence of v on secondary slip $\beta_{1}$.

**Figure 13:**
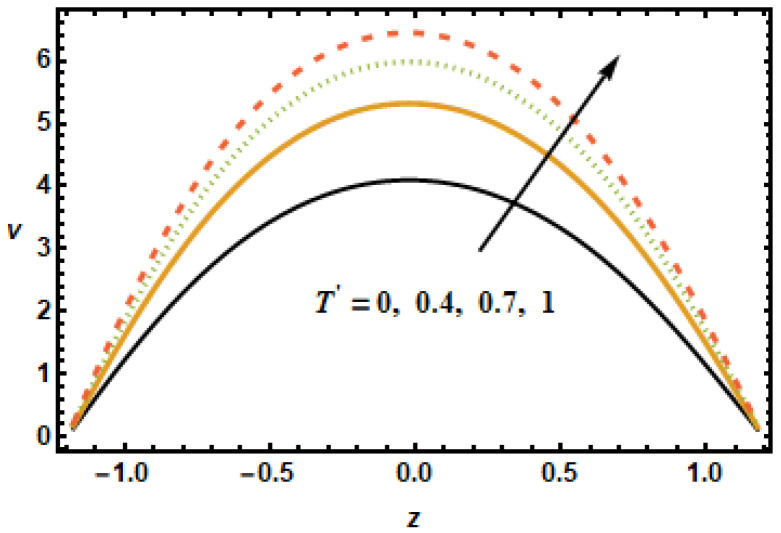
Dependence of v on rotation parameter $T^{\prime }$.

The dynamical effects of different parameters such as the Hartman number, the Hall parameter, the nanoparticle volume fraction, the permeability parameter, and the secondary slip parameter, and rotation parameter on the secondary velocity are given in Figure 8, Figure 9, Figure 10, Figure 11, Figure 12 and Figure 13. The decelerating effects of the Hartman number M, quantifying magnetic field strength, on the secondary velocity v are given in Figure 8. Consistent with the observations made in Figure 2, the secondary velocity also decreased with an increase in Hartman number *M* because of increased Lorentz force, which impeded the flow, as discussed above. Figure 9 illustrates velocity profiles generated for various values of the Hall parameter m to investigate the influence of the Hall parameter on secondary velocity. The results revealed that the Hall parameter had an accelerating influence on the flow, as seen by the increasing trend in velocities as the Hall parameter increased. By the decay of magnetic damping forces, the thermal conductivity of two-stage nanomaterial decreased, which resulted in higher secondary velocities.

Hall effect is necessary for the manufacturing of secondary velocity because m=0 means having no secondary velocity. To analyze the effects of nanoparticle volume fraction ϕ on the secondary velocity, we plotted velocity curves corresponding to different values of ϕ, shown in Figure 10. The results showed that the secondary velocity decreased for increasing values of ϕ. This is consistent with the physical effects associated with volume fraction ϕ, i.e., for higher values of volume fractions ϕ, the inter-particle interactions increased, resulting in a loss of energy. Figure 11 shows velocity curves plotted, corresponding to different values of the permeability parameter $K_{1}$. The velocity curves may be observed in the figure to have a rising tendency when the permeability parameter $K_{1}$ increased. The increment in fluid velocities resulted from decreased hindrance experience by the fluid at large values of permeability parameter $K_{1}$.

In Figure 12, we show results in terms of secondary velocity for several values of the secondary slip parameter. In agreement with the observations made in Figure 5, secondary velocity increased with increasing values of the slip parameter. As shown in Figure 5, the effect of the slip parameter was observed to be localized for secondary velocities as well. This sketch represents a very different trend compared to the other parameters. Figure 13 depicts the influence of the rotation parameter $T^{\prime }$ on secondary velocity. The secondary velocity rose as the rotation parameter grew larger, due to greater inertial forces, as shown in the displayed figure.

### 3.3. Temperature Analysis

**Figure 14:**
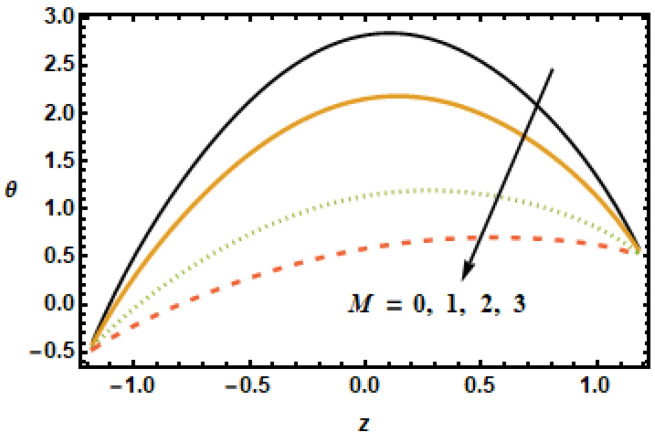
Effects of Hartman number M on θ.

**Figure 15:**
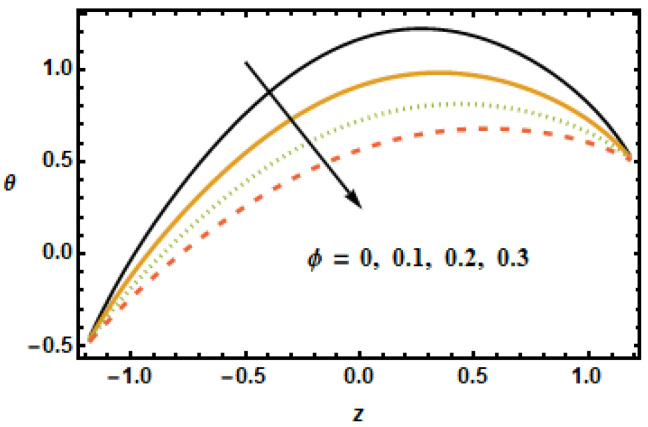
Effects of volume fraction ϕ on θ.

As discussed in the introduction, thermal properties of the base fluids could be considerably enhanced with the presence of nanoparticles. In this section, we explore the effects of the Hartman number M, the nanoparticle volume fraction ϕ, the permeability parameter $K_{1}$, the thermal slip parameter $\beta_{2}$ and the radiation parameter Rd on thermal characteristics of the flow. Figure 14 shows temperature profiles for diverse values of the Hartman number. As the values of the Hartman number increased, fluid velocities decreased, as shown in Figure 2 and Figure 8. This decrement in velocities resulted in less inter-particle interaction, due to which temperature profiles showed a decreasing trend with increasing values of the Hartman number. The effects of the volume fraction ϕ on the thermal profiles were investigated, as shown in Figure 15. The results showed a drop in temperature profiles at higher values of the volume fraction ϕ. For higher volume fractions, the effective viscosity of the mixture increased, due to which the thermal conductivity was reduced; hence, the temperatures dropped at large values of the volume fraction ϕ. The main point is that the absence of copper and the addition of nanoparticle volume fraction in base fluid resulted in increased thermal conductivity of the fluid; thus, by enhancing nanoparticles, the fluid capacity was increased to accumulate the consequent temperature fall.

**Figure 16:**
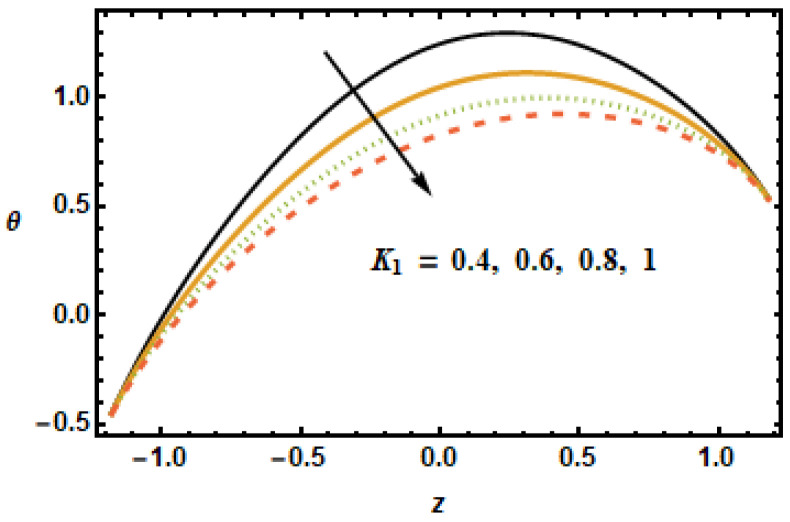
Dependence of θ on $K_{1}$.

**Figure 17:**
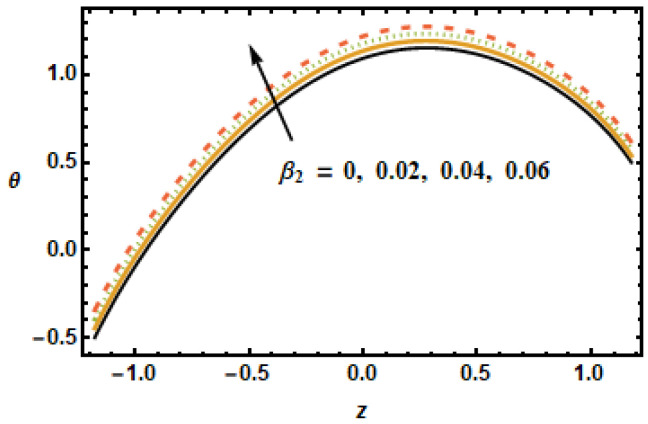
Dependence of θ on thermal slip.

**Figure 18:**
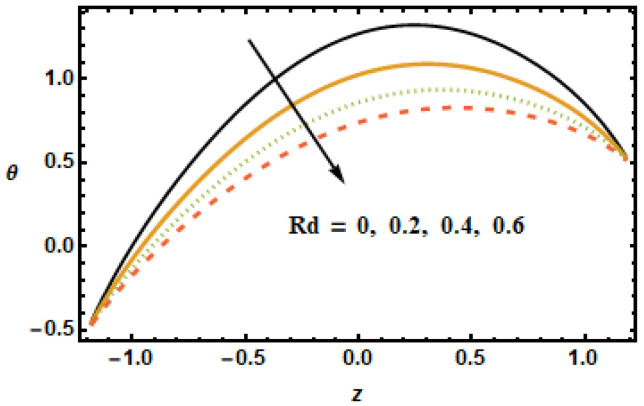
Effects of radiation Rd on θ.

The effect of the permeability parameter on temperature was observed. It can be observed in Figure 16 that temperature differences enlarged at the center of the graph. By increasing the porosity, the system leaves the temperature. Figure 16 shows temperature profiles at different porosity levels. Notice the decreasing trend in temperature curves corresponding to increased values of the permeability parameter. This is due to the fact that fluid experiences less resistance for large porosity. Notice that the two parameters, the Hartman number M and the volume fraction ϕ, have similar trends for velocities and temperature distribution, whereas permeability of porous medium shows different behavior for temperature compared to that of axial and secondary velocities. The effects of the slip parameter $\beta_{2}$ on the thermal characteristics of the fluid is shown in Figure 17. The plotted results show that the slippage condition enhanced the heat transfer rate. This is because, for large values of the slip parameter, the velocity gradients rose, which, in turn, enhanced inter-particle interactions, generating more heat. For the radiation parameter Rd, the temperature profiles showed a decreasing trend, as is depicted in the results plotted in Figure 18. The temperature in the inner domain lowered when the radiation parameter Rd increased because energy absorption reduced as the radiation parameter increased.

### 3.4. Entropy Generation Analysis

**Figure 19:**
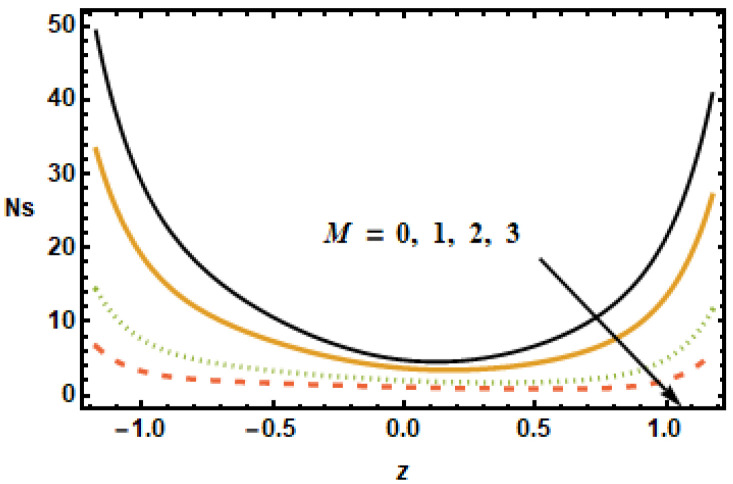
Effects of Hartman number M on Ns.

**Figure 20:**
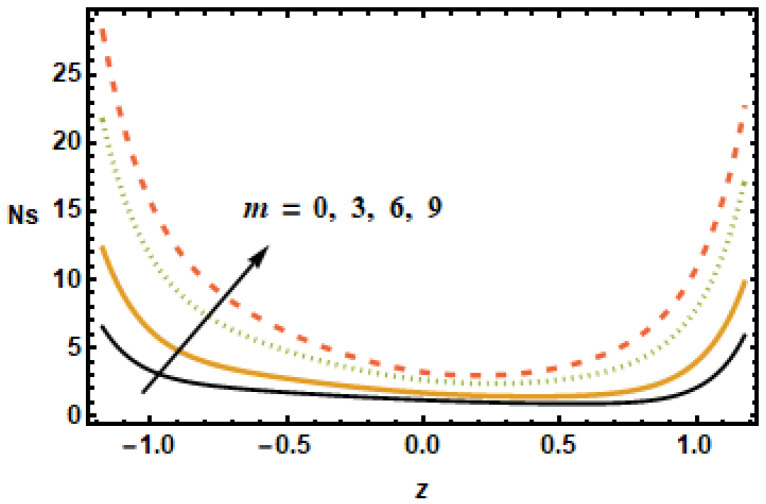
Effects of Hall parameter m on Ns.

**Figure 21:**
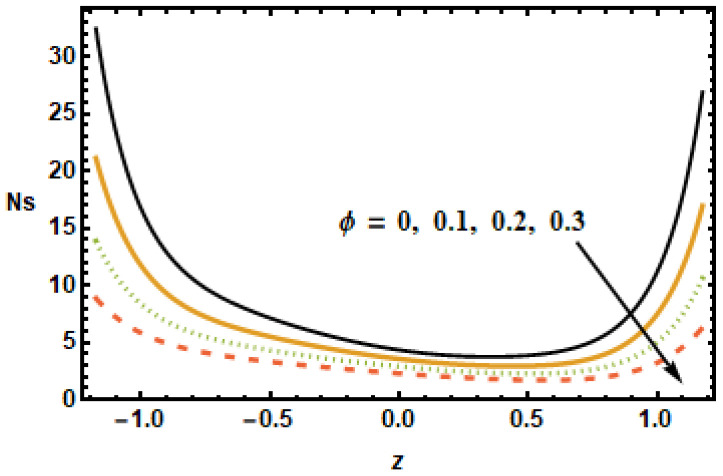
Effects of volume fraction ϕ on Ns.

**Figure 22:**
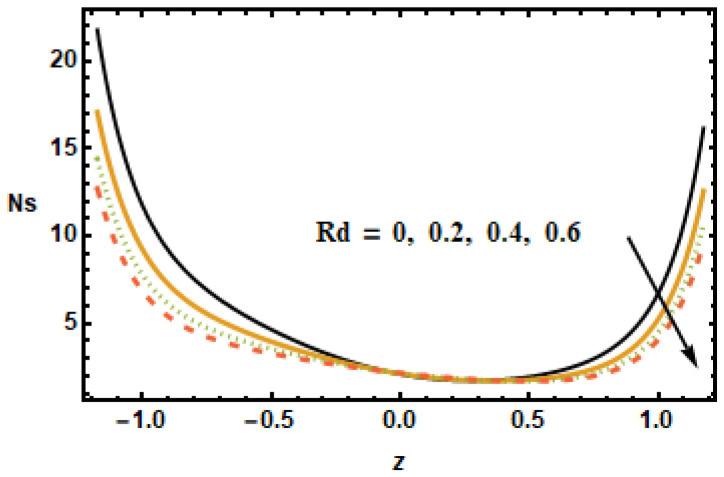
Effects of radiation parameter Rd on Ns.

**Figure 23:**
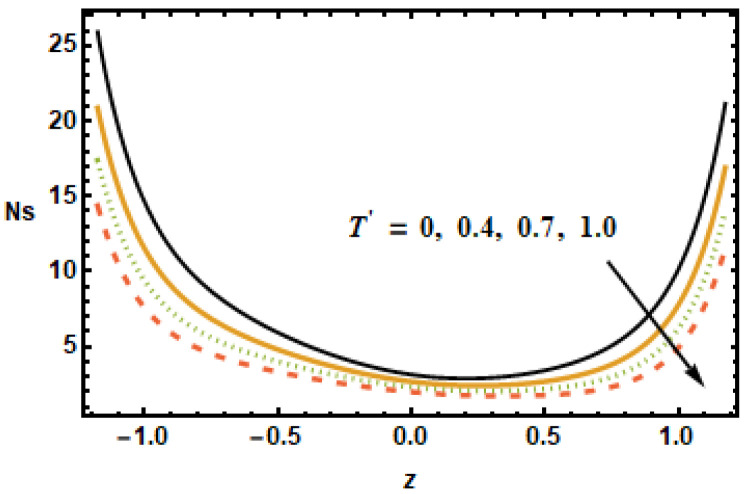
Effects of rotation parameter $T^{\prime }$ on Ns.

**Figure 24:**
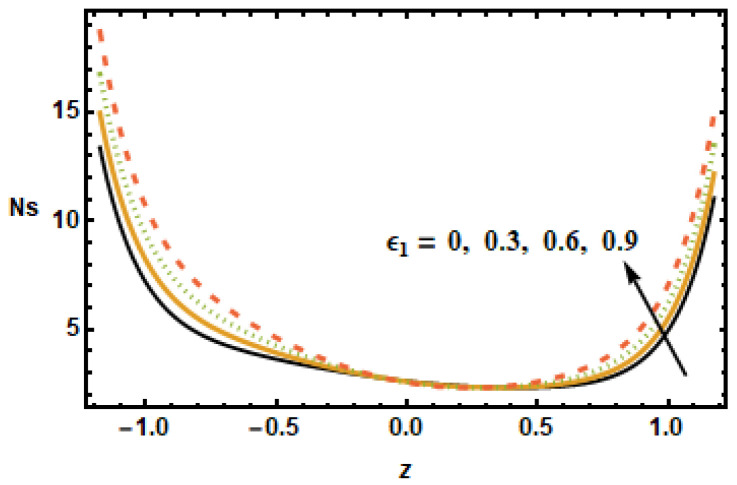
Effects of heat generation $\varepsilon_{1}$ on Ns.

The impact of entropy generation on different parameters, i.e., the nanoparticle volume fraction parameter, the radiation parameter, the Hall parameter, the heat generation/absorption parameter, the rotation parameter, and the Hartmann number (ϕ, Rd, m, $\varepsilon_{1},$ $T^{\prime },$ and M) is discussed in Figure 19, Figure 20, Figure 21, Figure 22, Figure 23 and Figure 24. The results plotted in Figure 19 show the entropy production number Ns for diverse values of the Hartmann number M. In the plotted results, the entropy generation number exhibited an inversely proportional association with the Hartmann number, i.e., Ns dropped as the value of M increased. Notice that, for diverse values of the Hartmann number, the difference between the entropy was maximum at the boundaries, whereas, in the center of the domain, this difference was the lowest. Figure 20 shows spatial variation of the entropy parameter Ns for dissimilar values of the Hall current parameter m. As shown above, velocity gradients increased at higher values of *m*; it is, therefore, seen in the figure that the entropy generation number Ns increased with *m*.

To understand the effect of the nanoparticle volume fraction ϕ on the entropy production number Ns, results were plotted for different volume fractions ϕ. It is noted that, by increasing the value of the nanoparticle volume fraction ϕ, the entropy generation number Ns decreased. Similarly for the radiation parameter Rd, the entropy generation number decreased due to small thermal gradients resulting from a loss of thermal energy due to radiation.

It is depicted in Figure 23 that Ns reduced for rising values of the rotation parameter $T^{\prime }$, which means that the entropy generation number increased in the absence of rotation. The results in Figure 24 show that, for large values of heat generation or the absorption parameter $\varepsilon_{1}$, Ns increased at the boundaries but remained constant at the center of the domain. Notice that the difference was minimal for smaller values of $\varepsilon_{1}$, evident from the observation that the black and yellow curves resided very close to each other. It is also worth noting that the entropy generation trend for all of the parameters showed a decreasing trend, with the exception of the heat generation/absorption parameter, which showed an increasing trend. Furthermore, the Hartman number, the volume fraction and the rotation influence axial velocity and entropy generation changed in a similar manner.

### 3.5. Bejan Number Analysis

**Figure 25:**
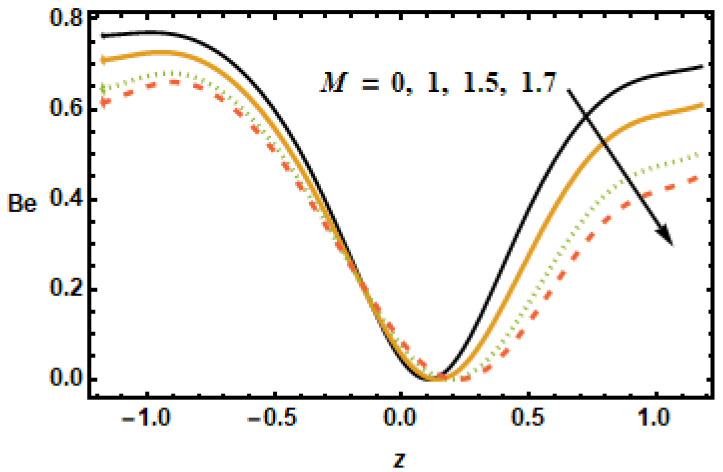
Effects of Hartman number M on Be.

**Figure 26:**
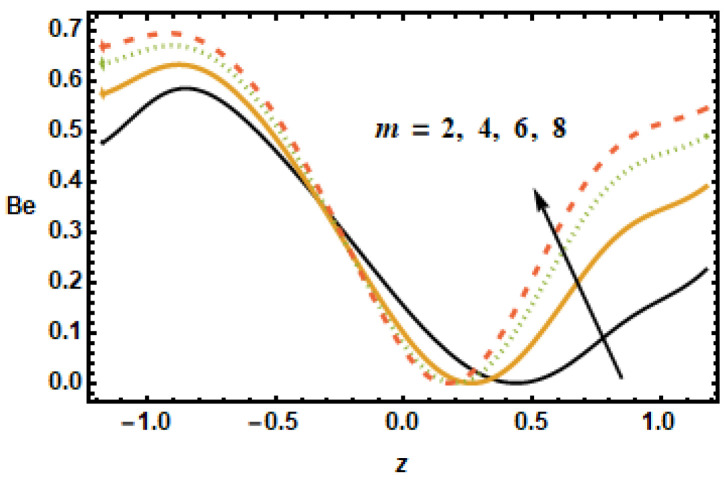
Effects of Hall parameter m on Be.

**Figure 27:**
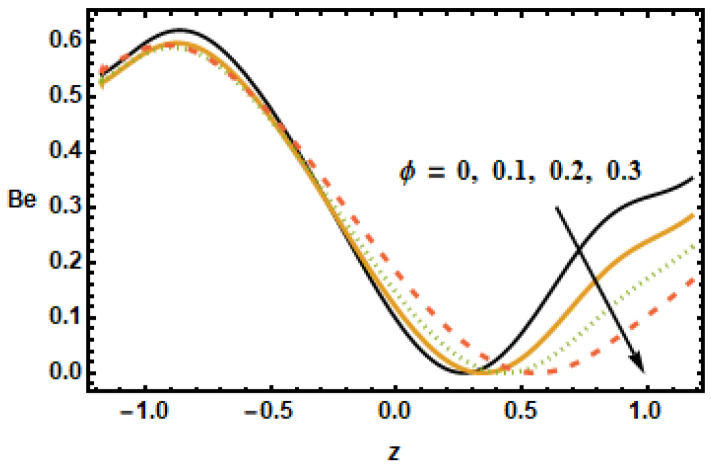
Effects of volume fraction ϕ on Be.

**Figure 28:**
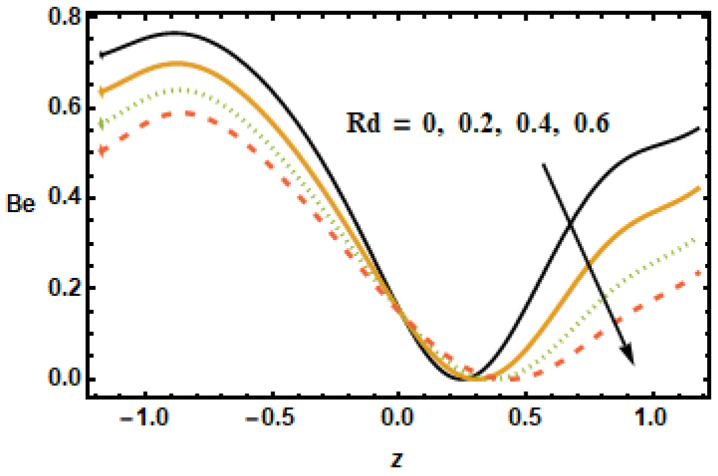
Effects of radiation parameter Rd on Be.

**Figure 29:**
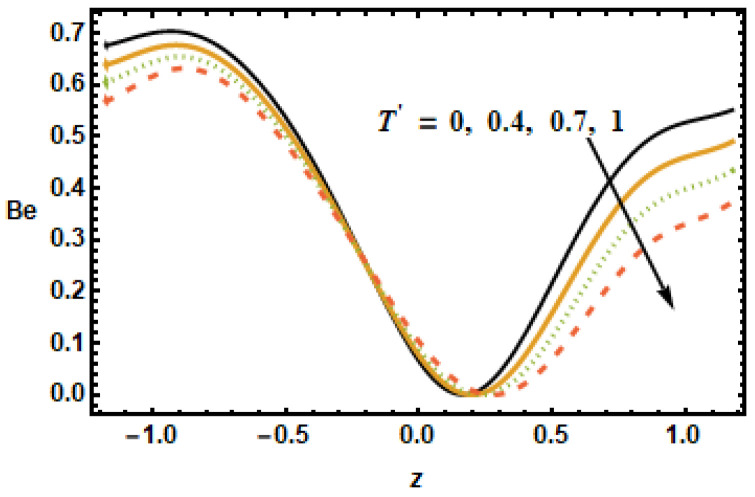
Effects of rotation parameter $T^{\prime }$ on Be.

**Figure 30:**
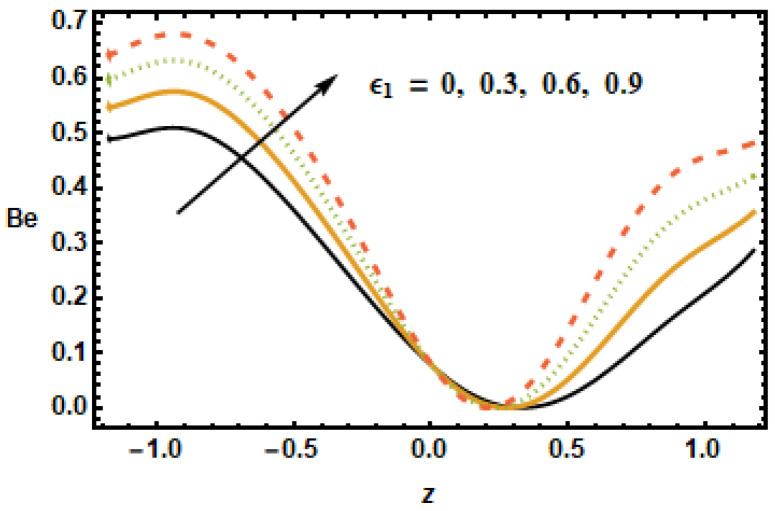
Effects of heat generation $\varepsilon_{1}$ on Be.

Figure 25, Figure 26, Figure 27, Figure 28, Figure 29 and Figure 30 are portrayed to depict the influence of the Bejan number Be on various parameters, including the heat generation/absorption parameter, the volume fraction, the Hall parameter, the rotation, the Hartman number and radiation. Figure 25 shows the inverse relation of the Bejan number against the Hartman number M, i.e., by increasing values of M across the channel, the Be values dropped. It is clearly seen that the Bejan number decreased at the starting and ending point, but it remained the same at the region from −0.5 to 0. Figure 26 shows that Be magnified across the boundaries with increasing values of the Hall parameter m but Be was reduced in the middle of the channel due to reduced strengths of the magnetic field, which reduced the fluid acceleration.

The plot in Figure 27 revealed that the Bejan number Be decelerated for greater values of nanoparticle volume fraction ϕ. It is so because of the irreversibility of heat transfer, total entropy generation is greater than entropy generation. At the center of the channel, the trend changes whereas at the right end of channel Bejan number is almost constant for increasing values of ϕ. In Figure 28, it is noted that the increasing value of the radiation parameter Rd resulted in a reduced Bejan number Be due to the fact that temperature gradients drop as the radiation parameter Rd increased.

Rotation parameter $T^{\prime }$ has similar behavior, such as the Hartman number M having an inverse relation with the Bejan number due to angular velocity across the boundaries of the channel. It is seen in Figure 30 that the heat flux parameter $\varepsilon_{1}$ increased the Bejan number Be along the boundaries, whilst the dependence remained constant in the center of the channel. The Bejan number had an increasing and decreasing trend for the Hall Effect and the volume fraction, respectively. Rotation, radiation and the Hartman number affected the Bejan number and entropy generation number in a similar manner. Furthermore, changes in the temperature distribution, entropy production and the Bejan number due to the Hartman number was similar.

## 4. Conclusions

The peristaltically moving flow of nanomaterial in a porous rotating channel with generalized complaint walls was investigated in this article. The impact of the magnetic field, the Hall parameter, the heat source/sink, thermal radiation, Joule heating, and boundary slip on the velocity and temperature profiles was discussed in detail. The major goal of this research was to look at how entropy generation and the Bejan number are affected by varying physical conditions. The following are the most important consequences:

- The axial velocity was inversely proportional to the Hartman number, the volume fraction of nanoparticles, and the rotation parameters. It decreased when these parameters were increased.
- The axial velocity increased when the porosity and first order slip parameters were increased.
- Secondary velocity decreased with the increase of the Hartmann number and the nanoparticle volume fraction.
- Increasing the Hall parameter, porosity parameter, secondary velocity slip parameter, and rotation parameter improved secondary velocity.
- The temperature profile was enhanced for only the thermal slip parameter, demonstrating temperature rises due to slip effects.
- The temperature dropped for increasing values of the Hartman number, the Hall parameter, porosity, and the radiation parameters.
- Increasing the Hall parameter and the heat generation/absorption parameter enhanced the amount of entropy generation.
- Entropy was reduced for large values of the Hartman number, the nanoparticles volume fraction, the radiation parameter, and the rotation parameter.
- The alternative behavior for the Hall parameter and the nanoparticles volume fraction was represented by the Bejan number.
- Due to the pressure drop throughout the length of the channel, the Bejan number rose in relation to the heat generation/absorption parameter.
- The Bejan number and entropy generation had the same behavior when it came to physical parameters such as the rotation parameter, the radiation parameter, and the Hartman number.

## Figures and Tables

**Table 1:** Expressions for thermal features of nanofluid Ali et al. [38].

Properties	Nanofluid
Density	$\rho_{nf}=\left(1-\phi \right)\rho_{f}+\phi \rho_{p},$
Viscosity	$\mu_{nf}=\frac{\mu_{f}}{{\left(1-\phi \right)}^{2.5}},$
Heat capacity	${\left(\rho c_{p}\right)}_{nf}=\left(1-\phi \right){\left(\rho c_{p}\right)}_{f}+\phi {\left(\rho c_{p}\right)}_{p},$
Thermal conductivity	$\frac{\kappa_{nf}}{\kappa_{f}}=\frac{\kappa_{p}+2\kappa_{f}-2\phi \left(\kappa_{f}-\kappa_{p}\right)}{\kappa_{p}+2\kappa_{f}+\phi \left(\kappa_{f}-\kappa_{p}\right)},$
Thermal expansion	${\left(\rho \beta \right)}_{nf}=\left(1-\phi \right){\left(\rho \beta \right)}_{f}+\phi {\left(\rho \beta \right)}_{p},$
Electric conductivity	$\frac{\sigma_{nf}}{\sigma_{f}}=1+\frac{3\phi \left(\frac{\sigma_{p}}{\sigma_{f}}-1\right)}{\left(\frac{\sigma_{p}}{\sigma_{f}}+2\right)-\phi \left(\frac{\sigma_{p}}{\sigma_{f}}-1\right)},$

**Table 2:** Numerical values of thermal properties of nanomaterial Ali et al. [37].

Physical Properties	Water ($H_{2}O$)	Copper (Cu)
$\rho \left(\mathrm{kg}m^{-3}\right)$	997.1	8933
$c_{p}\left(J{\mathrm{kg}}^{-1}K^{-1}\right)$	4180	385
$\kappa \left(Wm^{-1}K^{-1}\right)$	0.613	401
$\beta \left(K^{-1}\right)\times 10^{-5}$	21	1.67
$\sigma {\left(\Omega m\right)}^{-1}$	0.05	$5.96\times 10^{7}$

## Data Availability

Not applicable.

## Nomenclature

**List of Symbols**		**Greek Symbols**	
T	Fluid temperature (K)	ϕ	Nanoparticles volume fraction
$T_{0}$	Lower wall temperature (K)	ρ	Fluid density (kg m^−3^)
$T_{1}$	Upper wall temperature (K)	ψ	Stream function (m^2^ s^−1^)
$T_{m}$	Mean Temperature of nanofluid	μ	Dynamic Viscosity (kg m^−1^ s^−1^)
$c_{p}$	Specific heat (J kg k^−1^)	υ	Kinematic Viscosity (m^2^ s^−1^)
Ω	Angular speed	σ	Electric conductivity (Sm^−1^)
Ns	Entropy generation	κ	Thermal conductivity (W m^−1^ k^−1^)
*Be*	Bejan number	*R*	Dimensionless heat flux parameter
Pr	Prandtl number	$\varepsilon_{1}$	Heat generation/absorption parameter
*Ec*	Eckert number	δ	Wave number
*Br*	Brinkman number	**Subscript**	
M	Hartmann number	nf	Nanofluid
u, v, *w*	x,y,z velocity component (m s^−1^)	*F*	Base fluid
Re	Reynolds number	*P*	Particle of nanofluid
$B_{o}$	Strength of magnetic field (T)	*eff*	Effective
